# Selection signatures in the CIMMYT International Elite Spring and Semi‐arid Wheat Yield Trials

**DOI:** 10.1002/tpg2.20165

**Published:** 2021-11-08

**Authors:** Alexandre Mondaini, Umesh Rosyara, Deepmala Sehgal, Susanne Dreisigacker

**Affiliations:** ^1^ Institute of Plant Breeding, Seed Science and Population Genetics Univ. of Hohenheim Fruhwirthstrasse 21 Stuttgart 70593 Germany; ^2^ current address: Univ. of Hong Kong 21 Sassoon Rd, Pok Fu Lam Hong Kong; ^3^ International Maize and Wheat Improvement Center (CIMMYT), Global Wheat Program Km45 Carretera Mexico‐Veracruz Texcoco Edo. de México 56237 México; ^4^ current address: BASF 26 Davis Dr. Research Triangle NC 27709 USA

## Abstract

The International Maize and Wheat Improvement Center (CIMMYT) annually distributes advanced wheat (*Triticum aestivum L*.) breeding lines to collaborators worldwide through the International Wheat Improvement Network. Lines are disseminated through international nurseries, including the Elite Spring Wheat Yield Trial (ESWYT) targeted to optimal (irrigated and high production) wheat production areas and the Semi‐arid Wheat Yield Trial (SAWYT) targeted to low rainfall environments. A total of 2,184 wheat lines that formed the ESWYT and SAWYT since 1979 and 1992, respectively, were genotyped using genotyping‐by‐sequencing to explore trends of genetic diversity and selection footprints associated with continuous crop improvement and adaptation. Due to a small population size of each trial, adjacent year trials were pooled into subpopulations. Population structure was evaluated using discriminant analysis of principal components and fixation index. High levels of admixture within and across the ESWYT and SAWYT subpopulations were revealed, indicating that the entire genetic diversity in the overall CIMMYT germplasm pool is harnessed to target core traits to individual mega‐environments. Genome wide scans of deviations of minor allele frequencies at each marker identified large linkage blocks in several chromosomes. The scans also revealed that 9.8 and 2.0% of the SNP markers could be associated to selection signatures over time and to environmental adaptation (significant deviations between ESWYT and SAWYT), respectively. Several known genes and previously identified haplotypes associated with grain yield in more recent CIMMYT elite germplasm did fall into genomic regions with directional selection.

## Core Ideas

- Genomic changes may be tracked across internationally distributed CIMMYT wheat lines.
- Structure analysis revealed admixture among elite lines targeted to different environments.
- Genome scans identified large linkage blocks in several chromosomes.
- A higher percentage of selection signatures resulted from selection over time than over environments.

**Abbreviations**
BIC: Bayesian information criteria
CIMMYT: International Maize and Wheat Improvement Center
DAPC: discriminant analysis of principal components
DArT: diversity array technology
ESWYT: Elite Spring Wheat Yield Trial
*F_ST_*
: fixation index
GBS: genotype‐by‐sequencing
GWAS: genome wide association studies
MAF: minor allele frequency
ME: mega‐environments
PC: principal component
QTL: quantitative trait loci
SAWYT: Semi‐arid Wheat Yield Trial
SNP: single nucleotide polymorphism

## 1 INTRODUCTION

Wheat (*Triticum aestivum L*.) is the most widely grown food crop in the world and the third most important in terms of global production after maize (*Zea mays* L.) and rice (*Oryza sativa* L.). Wheat is of fundamental importance in the human diet by accounting for about 20% energy and protein intake globally (FAO, 2016). Wheat is also a global commodity, being the most exported crop, countries/regions with the largest wheat production do not always correspond with those that have the highest wheat consumption (FAO, 2016). While the rate of world population growth is in general slowing down, it will still reach 8.6 billion people in 2030 and 9.8 billion by 2050 (van Bavel, 2013). Thus, meeting the global demand for wheat will require a substantial increase of grain yield production per unit area, which is currently around 3.3 t ha^‐1^ (FAO, 2017). Achieving this goal will be challenging as changing climate constraints pose risks not only to the harvested product directly but also hinder wheat growing area expansion.

The International Maize and Wheat Improvement Center (CIMMYT) annually distributes specific nurseries and yield trials as part of a system known as the International Wheat Improvement Network. Nurseries and trials are grown under local conditions across the developing world and the best candidates are selected for direct cultivar release or as parents for new locally made crosses (Baum et al., 2015; Reynolds et al., 2017). The data gathered by collaborators are catalogued, analyzed, and made available to the global wheat research community. Internationally recorded performance data are also crucial to select new parents for subsequent crosses and breeding. Among the annually distributed yield trials, the two most requested are the Elite Spring Wheat Yield Trial (ESWYT) for optimal (irrigated and high production) and the Semi‐arid Wheat Yield Trial (SAWYT) for low rainfall environments. Both trials target 45 million ha of wheat production area globally. The ESWYT started in 1979 and the SAWYT in 1992, thus having almost three decades of shared history.

Although studies on the phenotypic performance on the CIMMYT yield trials have been significant (Byth et al., 1976; L. A. Crespo‐Herrera et al., 2018; Leonardo A. Crespo‐Herrera et al., 2017; Crossa et al., 2007; DeLacy et al., 1993; Mondal et al., 2020), only a few genomic studies have been performed on these trials to date (Crossa et al., 2007; Dawson et al., 2013; Susanne Dreisigacker et al., 2012).

Signatures of selection are reflected by loci or regions in the genome that undergo changes through reduction and an increase or elimination of genetic variation due to natural or artificial selection. Such regions are changed due to their direct functional relevance or indirectly through their association with causative variants. These regions can shed light on evolutionary adaptation or breeding practices, by identifying changes of allele frequencies over a period that most likely involved relevant genes related to important adaptive and commercial traits (Afzal et al., 2019; Appels et al., 2018; Browning & Browning, 2016; Cavanagh et al., 2013; Laland et al., 2010; Neale & Kremer, 2011). Till date signatures of selection have not been studied in the CIMMYT wheat breeding germplasm. The objectives of this study were therefore to (a) unravel the genetic structures of the lines in the ESWYT and SAWYT yield trials genotyped with genotyping‐by‐sequencing (GBS) and (b) identify regions under selection on a genome‐wide basis.

**Core Ideas**

- Genomic changes may be tracked across internationally distributed CIMMYT wheat lines.
- Structure analysis revealed admixture among elite lines targeted to different environments.
- Genome scans identified large linkage blocks in several chromosomes.
- A higher percentage of selection signatures resulted from selection over time than over environments.

## 2 MATERIALS AND METHODS

### 2.1 Plant materials

We considered spring bread wheat lines included in the two historical international yield trials, ESWYT and SAWYT. In total, 2,184 lines were analyzed; 1,229 lines from ESWYT and 955 lines from SAWYT (Table 1, Supplementary Table S1).

**TABLE 1:** Summary of wheat lines included in two International Maize and Wheat Improvement Center international yield trials, the Elite Spring Wheat Yield Trial (ESWYT) and the Semi‐arid Wheat Yield Trial (SAWYT)

International yield trial	Time period in years	Number of trials	Average number of lines per trial	Replicated lines within each trial
ESWYT	1979–2016	38	32	323
SAWYT	1992–2016	25	40	191
Total	1979–2016	63	36	514
**Predefined subpopulations**				
ESWYT 1–7	1979–1985	7	28	58
ESWYT 8–13	1986–1991	6	27	69
ESWYT 14–25	1992–2003	12	46	129
ESWYT 26–38	2004–2016	13	49	74
SAWYT 1–12	1992–2003	12	44	105
SAWYT 13–25	2004–2016	13	47	79

A total of 514 lines were replicated over time, with 28 common lines between the two trials. Each individual trial consisted of about 30–50 lines. The ESWYT was first distributed in 1979 and has been continued ever since, while the SAWYT started in year 1992. Hence, since 1992 both ESWYT and SAWYT are being disseminated in parallel but are targeted to two distinct mega‐environments (ME) of which 12 were previously defined by CIMMYT (Braun et al., 1996). The ESWYT lines are targeted to ME1 defined as optimal (irrigated and high production) environments. The ME1 falls into winter temperate climatic areas with 36 million ha of low rainfall in Asia, Africa, and Mexico and has late summer heat stress. These areas are optimally irrigated and may suffer from specific diseases like rust and kernel bunt (*Tilletia indica*). The SAWYT lines are targeted to ME4, low rainfall environments. The ME4 are nonirrigated low rainfall areas that comprises globally of 12 million ha, which predominantly suffer from water stress. It is subdivided into three major sub‐ME: winter rain regions followed by late Mediterranean‐type drought (e.g., in Syria, Aleppo), early winter drought followed by late summer rain (e.g., in Marcos Juarez, Argentina) and regions that crop growth is highly dependent on soil‐stored moisture after monsoon rains (e.g., Dharwad, India) (Rajaram, 2002).

### 2.2 Genotyping

All 2,184 lines were genotyped using GBS (Elshire et al., 2011; Poland et al., 2012) to capture genome wide markers. Genotyping was performed on an Illumina HiSeq 2500 (Illumina Inc.) with each lane pooling 190 samples. Read length consisted initially of 100 bp, but after trimming for barcode removal, 64 bp sequence tags were derived. Single nucleotide polymorphisms (SNPs) were called using TASSEL 5 GBSv2 pipeline (Glaubitz et al., 2014) and anchored to the International Wheat Genome Sequencing Consortium reference sequence (RefSeq version 1.0). A total of 106,511 SNPs were reported, and three different filtering methods implemented by successive steps were applied at each SNP locus: (a) inbreeding coefficient ≥80%, (b) Fisher's exact Test (*p* < .001) and (c) chi‐square (expect inbreeding of 96%). Single nucleotide polymorphisms that passed at least one of the filters were recovered with a total of 82,697 SNPs retained. SNPs passing one (a), two (a and b), or three (a, b, and c) filters were 17,603, 34,998, and 30,096, respectively. Missing marker data was imputed with Beagle version 4.1 (Browning & Browning, 2016) by performing linear interpolation of ungenotyped variants. Markers with greater than 80% missing values and less than 5% minor allele frequency (MAF) were also removed.

### 2.3 Subpopulations

**FIGURE 1:**
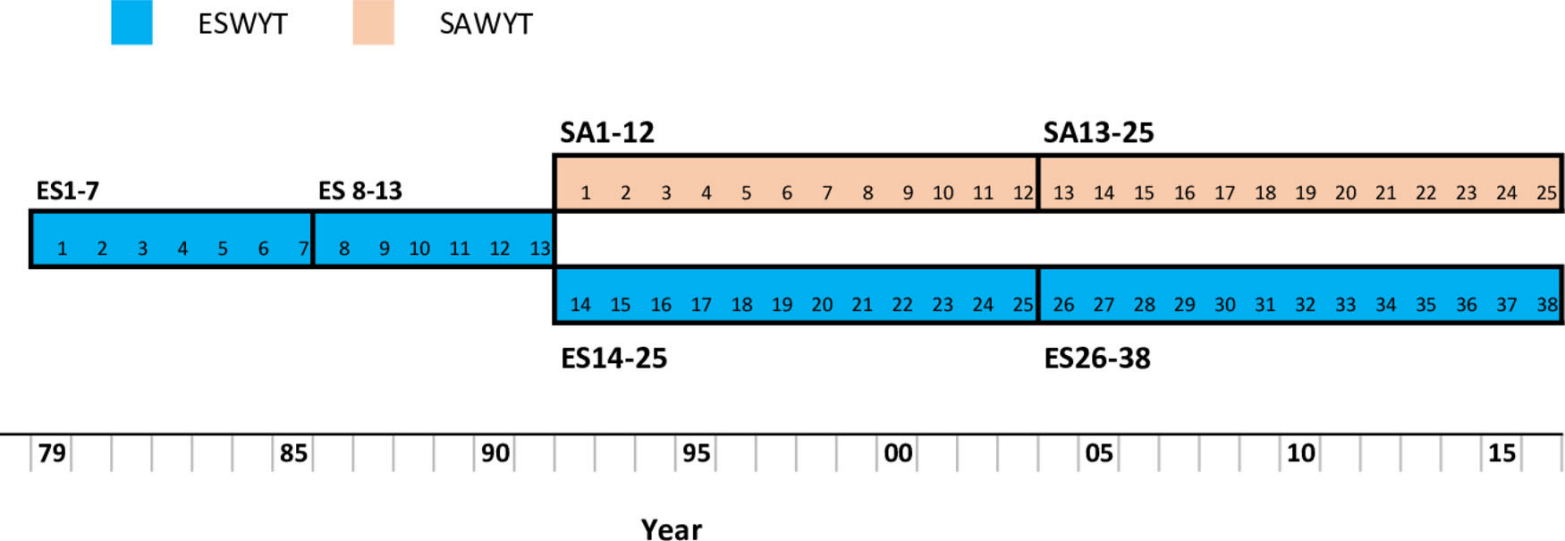
Timeline of predefined subpopulations. ESWYT, Elite Spring Wheat Yield Trial; SAWYT, Semi‐arid Wheat Yield Trial

As each individual yield trial was small with only 30–50 individuals, we pooled adjacent year trials into overall six subpopulations (Figure 1). From 1979 to 1991 only ESWYT was disseminated. We divided this time‐period into two subpopulations from year 1979 to 1985 (ES1–ES7) and from year 1986 to 1991 (ES8–ES13). Starting in 1992 both yield trials, ESWYT and SAWYT, were distributed in parallel. Similarly, we divided each yield trial distributed across this time‐period (1992–2016) into two subpopulations, from 1992 to 2003 (ES14–25, SA1–12) and from 2003 to 2016 (ES26–38, SA13–25). The two subpopulations of the initial germplasm pool of the ESWYT from 1979 to 1991 comprised a shorter year span than the later subpopulations (6 and 12 yr), to explore their relationship to the SAWYT, which expected to derive from the last years of the ESWYT (ES8–ES13).

### 2.4 Population structure

Population structure analysis was performed with R package ‘adegenet’ v.2.1.1 (Jombart & Ahmed, 2011), using discriminant analysis of principal components (DAPC). Discriminant analysis of principal components requires prior groups/populations to be defined, thus we implemented two approaches to look for these groups/populations: (a) clustering by unsupervised learning k‐means algorithm (Hartigan & Wong, 1979) and (b) supervised learning with predefined six subpopulations described in the previous section. When estimating clusters by k‐means, the maximum number of discoverable clusters was set to k = 40, with the number of randomly chosen initial centroids in each run set to *n* = 25 to better provide algorithmic convergence. The result of all sequential k clusters was then compared with the Bayesian information criteria (BIC) (Supplementary Figure S1), which should be lowest for the number of k or having the biggest decrease in BIC, thus the final number of clusters was set to k = 6. When visually comparing plots generated by both approaches, labeling populations a priori yielded better results of population differentiation, hence our preferred method. Discriminant analysis of principal components, as with principal component analysis, is a method of dimensionality reduction but in contrast focuses on optimizing the between group variance, whereas principal component analysis looks at the global variance often overlooking differences between groups. It accomplishes that by seeking discriminant functions (synthetic variables) that are linear combinations of the original variables (alleles). Discriminant analysis of principal components first converts the raw genotypic data into a standard principal component (PC) matrix, requests a prior number of principal components to be retained, and then creates the discriminant functions on the retained PCs. However, a trade‐off must be taken when choosing the number of PCs to be retained. While too few PCs would cause a reduction on the statistical power of discrimination between groups, too many can cause over‐fitting. Thus, we computed an alpha‐score optimization statistic that represents the proportion of successful reassignments of observed discriminant functions to values obtained by randomizing groups, in this manner we could determine the best number of PCs to be retained which was set 216.

### 2.5 Fixation index

To further explore population differentiation, we defined the fixation index (*F_ST_*) for the two yield trials. *F_ST_* is one of the most widely used descriptive statistics in population and evolutionary genetics (Holsinger & Weir, 2009). It provides a measure of genetic differentiation and is directly related to the variance of allele frequency among populations and, conversely, to the amount of similarity among individuals within populations. *F_ST_* values were calculated according to (Nei, 1988) using the R package ‘hierfstat’ v.0.04‐22 (Goudet, 2005).

For the *F_ST_* comparisons, we further divided the ESWYT and SAWYT subpopulations into 3‐to‐4‐yr groups from its primary until its most recent trials (e.g., ES1–4, ES5–7, ES8–10, ES11–13, ES14–16, SA1–3, etc.). This was done to observe environmental and temporal trends within the subpopulations under study. We analyzed *F_ST_* values in three ways:

1. by calculating *F_ST_* relative to the primary four‐year group (ES1‐4) to obtain genetic differentiation over time (e.g., between ES1–4 and ES5–7, ES1–4 and ES8–10, ES1–4 and ES11–13, etc.);
2. by calculating *F_ST_* between temporally consecutive groups to derive potential genetic shifts over a shorter time period (e.g., between ES1–4 and ES5–7, ES5–7 and ES8–10, SA1–3 and SA4–6, etc.); and
3. by calculating *F_ST_* between parallel groups of ESWYT and SAWYT trials to explore the trends of genetic differentiation between the two yield trials (e.g., SA1–3 and ES14–16).

### 2.6 Signatures of Selection

Minor allele frequency was computed for each SNP marker in the six subpopulations using the R package ‘hierfstat’ v.0.04‐22 (Goudet, 2005). The MAF values were plotted against physical marker position for each chromosome using R package lattice v.0.20‐38 (Sarkar, 2008).

Allelic frequency changes in temporal dimension were calculated as deviations of MAF from the primary subpopulation (ES1–7) to all others. A SNP marker was declared undergoing significant directional selection by applying a threshold of 1% quantile from both tails of the distribution of MAF deviation values. Markers under selection derived by environmental adaptation (SNP markers selected in different directions in the ESWYT and SAWYT) were explored by the deviation of MAF of each SNP from the two most recent subpopulations of each different yield trial (ES26–38; SA13–25) and applying the same percent quantile threshold. To further explore our results, we plotted several genes known to have historical relevance in CIMMYT germplasm into the genome‐wide scans and exploited the shifts in allele frequency of recently identified quantitative trait loci (QTL) consistently associated with grain yield (Sehgal et al., 2020). The QTL were detected based on a large haplotype‐based genome wide association study (GWAS) that included more than 6,000 CIMMYT advanced lines, evaluated in elite yield trials across five environments, in the last 7 years. The gene positions of the known genes were based on highest probability sequence alignment (BLASTn) of functional or closely linked gene‐based markers to the International Wheat Genome Sequencing Consortium reference sequence (RefSeq vs 1.0) performed in Ensembl Plants (https://plants.ensembl.org/Triticum_aestivum/Info/Index). Estimation of the frequency changes of the recently discovered QTL was possible because the same genotyping platform was used in both studies. The favorable allele of the first SNP of each haplotype was used to examine the allele frequency change over time in each yield trial, separately.

### 2.7 Statistical analysis

All data processing and statistical analyses, otherwise specified in above, were implemented in R v.3.5.1 (R Development Core Team, 2018).

## 3 RESULTS

### 3.1 Genotypic data

A final number of 40,530 SNP markers remained after data filtering and imputation, which were spread across all 21 chromosomes (Supplementary Table S3). The largest number of SNPs were physically positioned in genome B and the lowest in genome D. Only 1.3% of the SNP markers could not be assigned to any chromosome. Chromosomes 2B, 6B, and 7A were the three most densely covered chromosomes with on average one marker per 0.23 Mbp, while chromosomes 4D, 5D, and 2D were the least densely covered chromosomes averaging one marker per 1.00 Mbp.

### 3.2 Population structure

**FIGURE 2:**
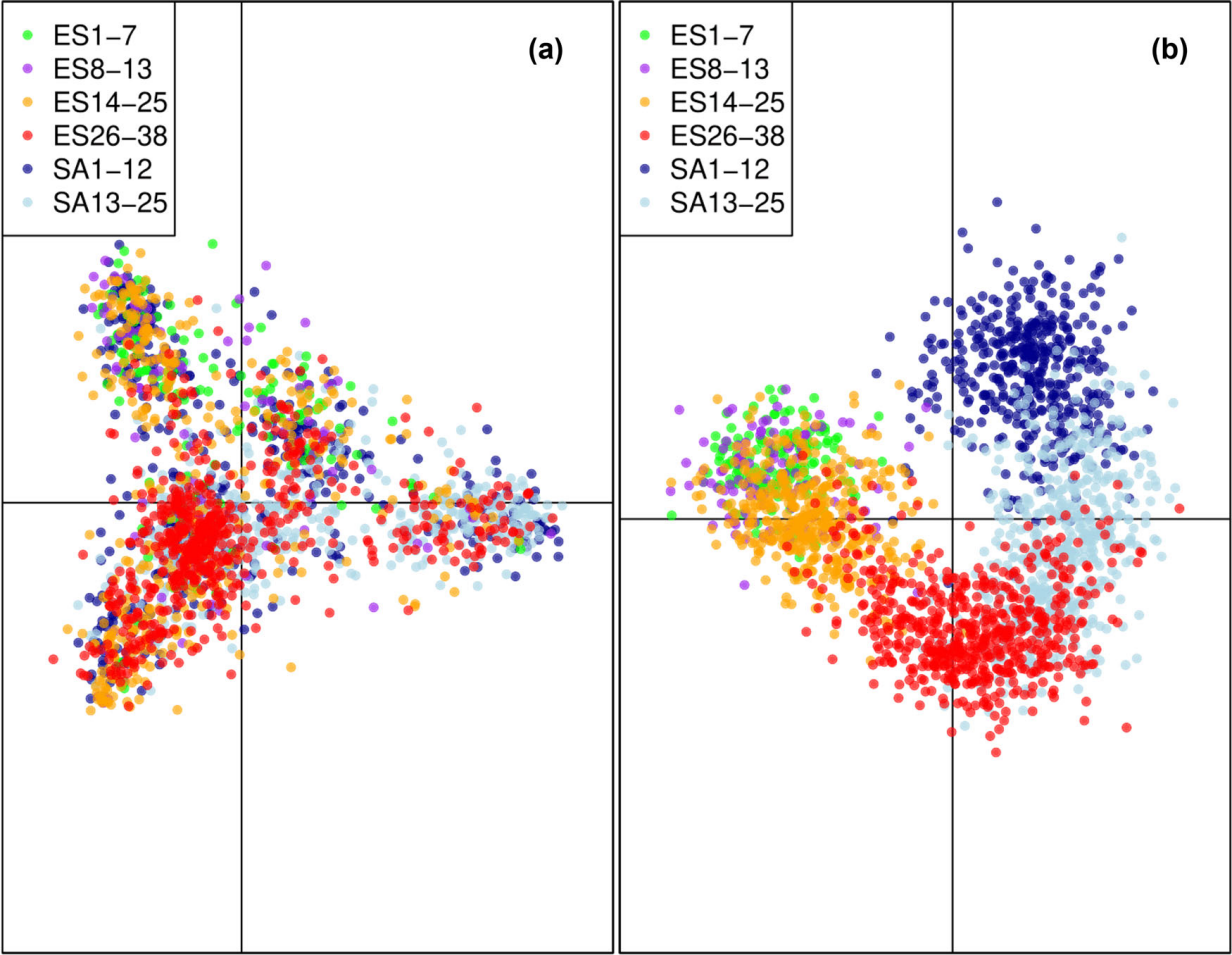
Discriminant analysis of principal components (DAPC) using two methods: (a) DAPC grouped by k‐means algorithm with color code superimposition for the predefined subpopulations; (b) DAPC grouped by predefined subpopulations

We superimposed the k‐means grouping structure by color‐coding every entry by its respective predefined subpopulation. The superimposition was done due to the unclear number of clusters to be chosen when analyzing the optimal number of k against the BIC (Supplementary Figure S1). The alpha‐score optimization statistic had its optimum at 216 PC (Supplementary Figure S2). All subpopulations showed admixture in the unsupervised DAPC (Figure 2a). However, the lines assigned to the first two subpopulations ES1–7 and ES8–13 tended to concentrate in the second quadrant of the DAPC plot and subpopulation ES26–38 tended to concentrate toward the third quadrant. The lines in subpopulation SA13–25 tended to group opposite to the previous subpopulations between the first and the fourth quadrant. Lines in the two subpopulations, SA1–12 and ES14–25, showed a random scatter in the plot without any tendency of grouping. Supervised DAPC showed that summarizing the data with predefined subpopulations provided an overall U‐shaped structure (Figure 2b). Like the unsupervised DAPC, the first two ESWYT subpopulations, ES1–7 and ES8–13, grouped closely together, next to subpopulation ES14–25. The ESWYT subpopulations formed the first half of the U‐shaped structure. The two SAWYT subpopulations formed the second half of the U‐shaped structure, while the subpopulation SA14–25 was related more closely to all ESWYT populations than the population SA1–12.

### 3.3 F_ST_

**FIGURE 3:**
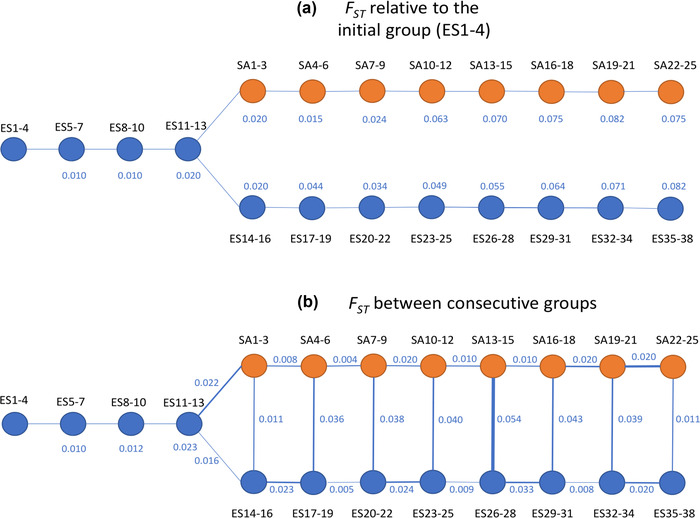
Fixation index (*F_ST_*) using two different approaches: (a) *F_ST_* values comparing each bin to the initial group; (b) *F_ST_* comparisons between consecutive groups

The *F_ST_* values between 3‐ and 4‐yr groups were overall low. The *F_ST_* values comparing each group to the initial group of four yield trials (ES1–4) increased over time for each yield trial (Figure 3a). While up to 2000 (SA7–9 and ES20–22), the *F_ST_* values relative to the initial group were always higher for the ESWYT than for the SAWYT, this relationship reversed after 2000.

The *F_ST_* values of consecutive groups were very similar in the ESWYT and SAWYT and ranged from 0.004 to 0.033. Comparisons between parallel groups of the two different yield trials from 1992 until 2016 showed increasing *F_ST_* values up to year 2006 (SA13–15 and ES26–28), which then declined (Figure 3b).

### 3.4 Changes in allele frequency over time and selection signatures

**FIGURE 4:**
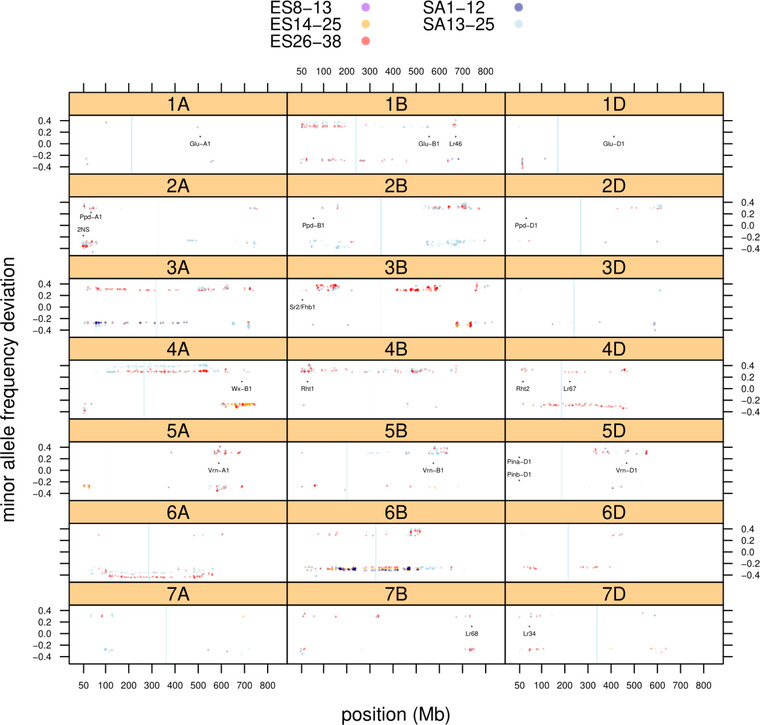
Temporal minor allele frequency (MAF) deviations, shaded areas are centromeric physical regions

**FIGURE 5:**
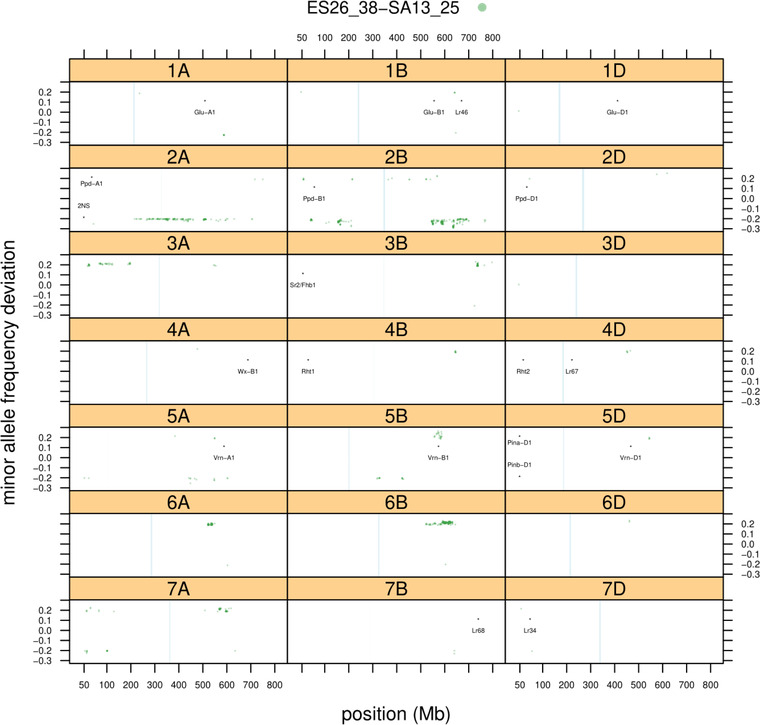
Environmental minor allele frequency (MAF) deviations, shaded areas are centromeric physical regions

We plotted all SNP markers based on MAF deviations to visualize patterns along the chromosomes (Supplementary Figure S3 and S4). Overall, we observed regions that showed large linkage blocks, specially flanking the centromere (on chromosomes 2A, 2B, 3D, 5A, 5B, 6A, 6B, and 7A). However, some large linkage blocks were also outside the centromeric region (e.g., on chromosome 1D). Such regions showed constant MAF values for each of the six subpopulations under study. Conversely, regions around the telomere showed a noisy pattern, most likely due to high rates of recombination. Density distribution plots of MAF deviations with their respective quantile boundary thresholds were devised for the different signatures of selection under study (Supplementary Figure S3). MAF deviations in temporal dimension had a rather smooth distribution that concentrated toward zero with the upper‐ and lower‐ boundaries of one percent quantiles beginning around 0.25 and −0.25 (Supplementary Figure S3a). The MAF deviations in environmental dimension (among the two yield trials) showed a peak shifted toward negative values, with the upper and lower boundaries beginning around 0.2 and −0.2 (Supplementary Figure S3a). From the total number of markers, 9.8% could be determined to be under selection over time following our threshold (Table 2). When considering the largest time span of MAF deviations (i.e., ES1–7 to ES26–38; SA14‐25), they accounted for almost all the temporal differences (8.2%) leaving only 1.6% to other subpopulations. Environmental differences accounted for 2.0% using the deviation from the two most recent subpopulations of each yield trial (ES26–38 and SA13–25). Most selection signatures derived from the MAF deviations of the first (ES1–7) to the two most recent populations (SA14–25; ES26–38), with 1,871 markers identified between ES1–7 and SA14–25 and 1,473 between ES1–7 to ES26–38, accounting together for 84% of the overall markers under selection. Chromosomes 6A, 6B, 4A, 4B, and 4D showed clear shifts of MAF deviation into one direction, while other chromosomes (e.g., 3A, 1B, 5A) showed allele frequency shifts into both directions. Allele frequencies shifts spanned substantial physical distances representing large linkage blocks, often (but not always) around the centromere (Figure 4). When considering MAF deviations of environmental adaptation, we detected 797 markers under selection. Chromosomes 3B, 1B, 5B, 2A, and 5A in descending order had the higher concentration of markers with a range of 48 to 159 (Figure 5). Chromosomes 2A, 2B, and 6B showed a larger number of markers concentrated in big linkage blocks.

**TABLE 2:** Summary of observed signatures of selection across International Maize and Wheat Improvement Center's the Elite Spring Wheat Yield Trial and the Semi‐arid Wheat Yield Trial

Selection signature type	Numbers of markers (%)	Populations
Over time	3,975 (9.8%)	ES1–7 – all
Over largest time span	3,344 (8.2%)	ES1–7 – ES26–38; SA13–25
Over environments/trials	7,97 (2.0%)	ES26–38 – SA13–25

**FIGURE 6:**
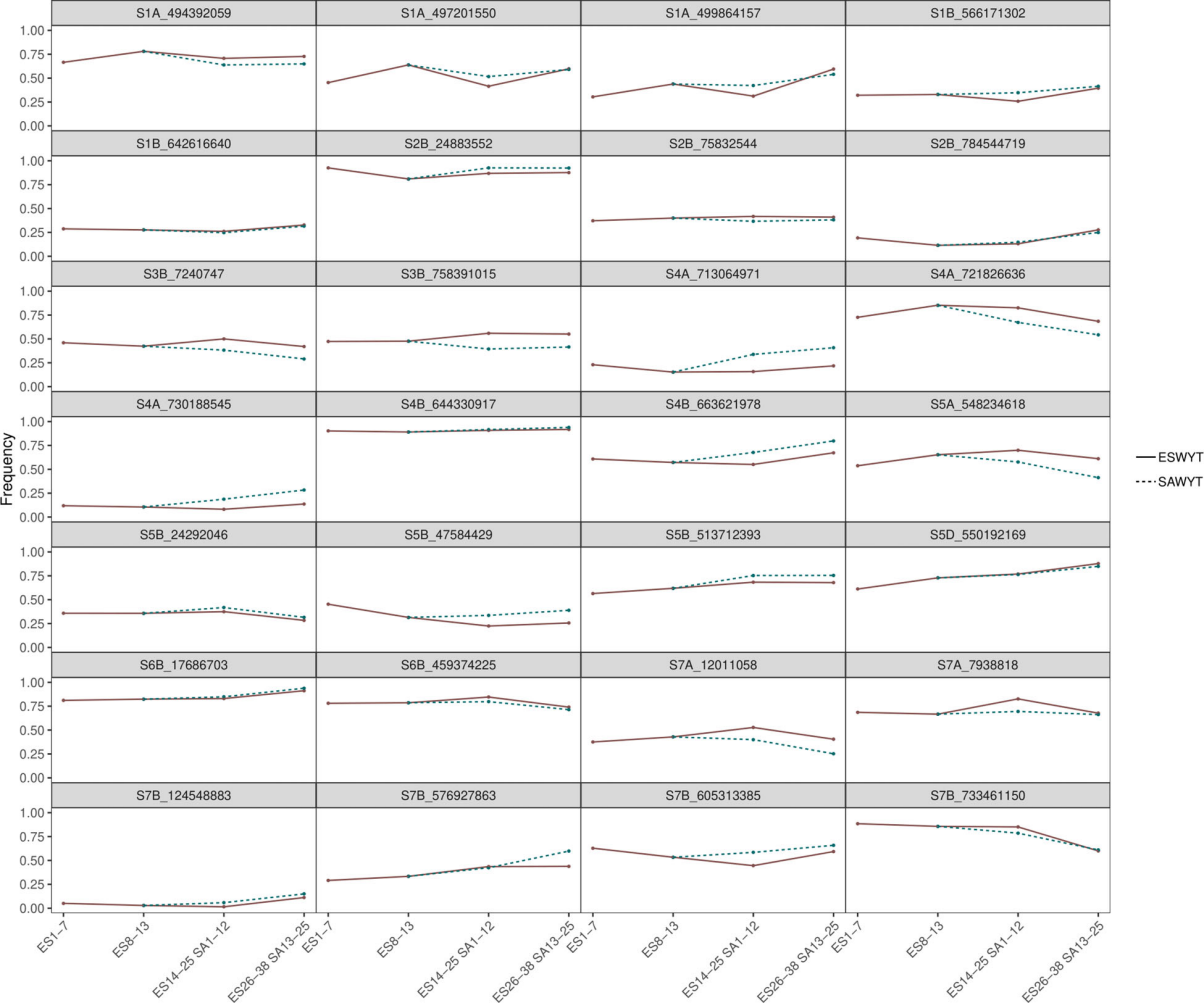
Favorable alleles over time of 28 grain yield quantitative trait loci (QTL). ESWYT, Elite Spring Wheat Yield Trial; SAWYT, Semi‐arid Wheat Yield Trial

### 3.5 Exploring known genes and QTL within the genome‐wide scans

We integrated several known genes and recently identified grain yield QTL in our genome‐wide scans to test if any of the genes fall into signatures of selection. On chromosome 1, the *Glu‐A1*, *Glu‐B1*, and *Glu‐D1* genes were flanked by only few markers. In contrast, gene *Lr46* located on chromosome 1B conferring slow‐rusting resistance was located within a small linkage block on temporal dimension with maximized allele frequency shifts in ES26–38 and SA13–25. On chromosome 2, the photoperiod response gene *Ppd‐A1* and the 2NS translocation introgressed from *Triticum ventricosum* (Tausch) Cess. carrying several disease resistance genes were flanked by linkage blocks in the temporal selection. Both loci showed a unidirectional selection pattern toward populations ES26–38 and SA13–25. On chromosome 3, the two closely linked disease resistance genes *Sr2* and *Fhb1* were only sparsely covered with markers. On chromosome 4, gene *Wx‐B1* was positioned within clear linkage blocks when in temporal dimension. The plant height genes *Rht1* and *Rht2* were located within linkage blocks with decreasing minor allele frequency over time. The rust resistance gene *Lr67* on chromosome 4D fell into a larger linkage block with selection in the opposite direction of *Rht2*. On chromosome 5, the vernalization response gene *Vrn‐A1* showed a bidirectional shift of the minor allele, while the minor alleles of markers under selection close to *Vrn‐B1* and *D1* decreased. The gene *Vrn‐B1* showed a clear sweep among environments. The grain hardiness genes *Pina‐D1*, *Pinb‐D1* had low coverage. On chromosome 7, markers flanking the two rust resistance genes, *Lr68* and *Lr34*, showed an increase of the minor allele over time.

Of the 28 grain yield QTL considered, 14 QTL showed an overall increase of the favorable allele over time for both yield trials (see Figure 6). In the ESWYT, the favorable allele of 19 (67%) QTL were increasing, while for nine (33%) QTL the favorable allele frequency decreased. In the SAWYT, the favorable allele of 16 (57%) QTL were increasing, while for 12 (43%) QTL the favorable allele frequency decreased over time. Some QTL were clearly selected; for example, QTL ‘*S5D_550192169*’ progressed from a frequency of 0.6 for the favorable allele to 0.9 in both most recent subpopulations. Other QTL were clearly selected in the opposite direction; for example, ‘*S5B_47584429*’, which departed from 0.45 frequencies in the most ancient population to 0.25 in the most recent.

## 4 DISCUSSION

The CIMMYT Global Wheat Program aims to develop better adapted wheat cultivars for farmers in the developing world. The International Wheat Improvement Network is thereby crucial in disseminating CIMMYT wheat lines carrying core traits of high and stable grain yield, disease resistance, and quality to national partners, thus representing a unique delivery pathway for new potential cultivars (Reynolds et al., 2017). The yield trials utilized in this study are disseminated to two out of 12 defined MEs, ME1 and ME4, which cover a large wheat production area globally (Rajaram, 2002). We genotyped the lines included in ESWYT and SAWYT since their initial distribution and investigated their genetic structure as well as signatures of selection within and among the trials.

### 4.1 Population stratification

Genotyping‐by‐sequencing is a simple highly multiplexed system for constructing reduced library representations (Elshire et al., 2011). It provides large numbers of SNPs for use in genotyping (Beissinger et al., 2013) while keeping the costs low, reducing sample handling, and providing efficient barcoding without reference sequence limits. With GBS, marker discovery and genotyping occur simultaneously, resulting in minimum ascertainment bias. Therefore, we had an extensive coverage of the wheat genome compared with previous studies that used different marker types and significantly less markers (Balfourier et al., 2007; S. Dreisigacker et al., 2004; White et al., 2008).

Population stratification is often driven by pedigree and individuals are rarely drawn from panmictic populations. We observed considerable admixture across the lines included in the ESWYT and SAWYT. This result supports previous findings on population stratification analysis in CIMMYT wheat germplasm. For instance, Dreisigacker et al. (2012) looked at the ESWYT and found instability using diversity array technology (DArT) markers and the software STRUCTURE on determining that the optimal number of K subpopulations also suggested strong relatedness of ESWYT germplasm. Another study using the same DArT marker technology and software determined the population stratification within individual ESWYT trials and found an overall pattern of unstructured populations (Crossa et al., 2007). More recently, Juliana et al. (2018) performed a principal component analysis using GBS and found no strong population structure among lines in CIMMYT elite yield trials. In contrast, clear population structure is observed in other breeding populations; for example, Canadian durum wheat (*Triticum durum* Desf.) (N'Diaye et al., 2018) with clear differences in pedigree and breeding program sources in Canada involving relatively little introduction of new material. Similarly, clear clusters were observed across 185 commercially released cultivars across the Great Plains in the United States (Ayalew et al., 2020). The CIMMYT is known for the consistent use of diverse parents and substantial genetic resources (e.g., landraces and synthetic hexaploid wheat) that have been vital to meet the requirements of global adaptation and enable the adoption of newly developed lines by cooperating wheat research programs globally (Dreisigacker et al., 2004; Rosyara et al., 2019). Hence, our results of substantial admixture in subpopulations were expected. However, some trends were also visible in our DAPC plot. Wheat lines from the later ESWYT and SAWYT tended to group apart from the first subpopulation including the initial 13 years of ESWYT only (from 1979 to 1992). In addition, lines specific to the later ESWYT and SAWYT concentrated in larger parts in different clusters.

Admixture of subpopulations in the ESWYT and SAWYT trials were also reflected by the overall low *F_ST_* values (0.01 < *F_ST_* < 0.05). The *F_ST_* can be thought of as a measure of the correlation of genes between populations. Low *F_ST_* values indicate gene flow between populations, which is expected in breeding materials as parent and subsequently their offspring are re‐cycled with every breeding cycle. Higher *F_ST_* values may arise by genetic drift or artificial selection (Suzuki, 2010). The increasing *F_ST_* values relative to the initial 4‐yr group suggests continued line improvement over time through artificial selection (Figure 5). Allele frequency shifts for both ESWYT and SAWYT on trends of genetic gains have been investigated in earlier studies by phenotypic data analysis. Crespo‐Herrera et al. (2018) estimated grain yield gains over eight years of ESWYT, 2006–2007 (ES27) to 2014–2015 (ES34) relative to the widely grown cultivar ‘Attila’ (GID14337) and found 1.67% improvement over time. Crespo‐Herrera et al. (2018) looked at grain yield improvement in SAWYT germplasm grown during 2002–2003 (SA11) to 2012–2013 (SA23) relative to four drought‐tolerant wheat lines used as constant checks and found an overall rate of increase of 1.8% in low yield environments and 1.4% in medium yield environments. The lower frequency shifts in the SAWYT from its initiating year in 1992 to 2000 together with the rather random scatter of the same lines in the DAPC plot, probably reflects the time that was required to establish the core traits and distribute the germplasm to ME4 (Trethowan et al., 2002). Among trials, *F_ST_* values increased until 2006 (ES26–28; SA13–15) and decreased from 2007 until 2016. During this period, established, large‐scale, and simultaneous testing pipelines for grain yield potential under several environmental conditions, including both, drought stress, and fully irrigated, were routinely performed. Substantial spillover was identified, for example, advanced lines targeted to ME1 were recognized as suitable parents to be used in ME4. Consequently, advanced lines are currently extracted from one larger diverse gene pool with specific emphasis on good performance across trial and management conditions, to maintain yield stability, a key feature of CIMMYT wheat germplasm and specifically important to cope with interannual variability in the yield driven by climate change (Morton, 2007).

### 4.2 Selection signatures

Loci under directional selection are expected to have higher interpopulation variability than intrapopulation variability when compared with neutral loci. Applied to our study, loci showing larger amounts of differentiation between subpopulations may reveal genomic regions that have been subject to diversifying selection. This differentiation can be assessed by patterns of variance in allele frequency or by examining *F_ST_* values between populations (Kirk & Freeland, 2011; Konijnendijk et al., 2015). A significant amount (9.8%) of loci indicated strong evidence of selection over 38 yr of breeding in CIMMYT's bread wheat elite lines. N'Diaye et al. (2018) explored loci under selection in breeding for durum wheat through three different methods: using the total variance *F_ST_*‐based outlier detection method (Lositan), the hierarchical island model (Arlequin), and the Bayesian genome scan method (BayeScan). Although breeding programs and species cannot be compared directly, the authors found a similar percentage of SNPs under selection. The percentage of SNPs under selection was higher over time than over trials/environments. This may be due to several reasons: (a) a longer time period when estimating selection signatures in temporal (38 years) versus environmental dimension (25 yr); (b) the above‐mentioned spillover effect, so genes conferring fitness to, for example, high yields or disease resistance, are similar in both sets, ESWYT and SAWYT, thus genes conferring fitness to one ME may confer fitness to several MEs; (c) both yield trials are targeted to different ME's, however these are assembled to maintain a broad genetic base in CIMMYT germplasm; (d) adaptation to a specific ME is expected to be based on a rather limited number of specific genes, thus a lower percentage of SNP under selection is expected. To the best of our knowledge, this study is the first that define gene regions driving adaptation to two MEs in wheat. A more detailed scrutiny of these regions will shed light on the genetic architecture of key adaptive traits in CIMMYT spring bread wheat elite lines.

### 4.3 Known genes and recently observed QTL

Several genes and QTL were observed as having physical proximity to the loci we have identified being under selection over time and through environmental adaptation. Physical proximity relevance on chromosome 1 can be attributed to gene *Lr46* which is known to confer slow‐rusting resistance and was first described by (Singh et al., 1998) in cultivar ‘Pavon 76’. On chromosome 2, the photoperiod sensitivity gene *Ppd‐A1* had closer proximity to a cluster of markers that were both present in our temporal selection signatures. Photoperiod insensitivity is being targeted by CIMMYT since N. E. Borlaug implemented the shuttle breeding concept (Rajaram, 2002). Almost all CIMMYT elite lines are photoperiod insensitive (showing high frequency of *Ppd‐D1a* allele), due to selection in two photoperiod contrasting environments during the shuttle. The 2NS translocation introgressed from *Ae. ventricum* and carrying at least three disease resistance genes (*Lr37*, *Yr17*, *Sr28*) were flanking several markers also on chromosome 2. Cultivars with the CIMMYT line ‘Milan’ in their pedigree are known to contain the 2NS translocation, which recently also showed high levels of resistance to wheat blast under field conditions (Cruz et al., 2016; Juliana et al., 2018). Interestingly the same genomic region was also identified as selection sweeps in the studies by (Ayalew et al., 2020; Dadshani et al., 2021). On chromosome 4, the waxy gene *Wx‐B1* was physically located close to markers under temporal selection. Also, dwarfing genes *Rht1* and *Rht2* were under temporal selection. All CIMMYT lines today are semi‐dwarf and carry, in comparison with the early green revolution lines mainly the dwarfing allele at *Rht1*, some the dwarfing allele at *Rht2*. On chromosome 5B, all three homologous vernalization genes were under temporal selection and *Vrn‐B1* was also under environmental selection. These results were in line with CIMMYT internal trait‐based marker data related to various phenology genes, which showed an overtime increasing frequency of the spring alleles at *Vrn‐B1* and *Vrn‐D1* and of the winter allele at *Vrn‐A1*, specifically in the SAWYT. On chromosome 7, the slow‐rusting resistance gene *Lr34* and the adult‐plant resistance gene *Lr68* were in proximity of markers selected in temporal dimension, emphasizing CIMMYT efforts on providing durable rust resistance.

A logical next step would be to relate the selection signatures to several key phenotypic traits. However, phenotypic data (especially from the initial years) have shown to be highly unbalanced. Therefore, we plotted allele frequency changes of recent GWAS results for grain yield derived from an extensive data set of CIMMYT advanced lines (Sehgal et al., 2020). In both yield trials, for more than half of the QTL (57% and 67% for the SAWYT and ESWYT, respectively) the favorable allele increased in frequency over time. This tendency corroborates the trait relevance of the identified QTL. Most importantly, an important genomic region on chromosome 5D was identified, which was under strong selection. This genomic region that showed an increase in frequency over time in trials was also identified by Sehgal et al. (2020) using haplotype‐based GWAS. Monitoring and additional pyramiding of these QTL offer breeders new opportunities for further improvement facilitating marker‐assisted or genomic selection.

## AUTHOR CONTRIBUTIONS

Alexandre Mondaini: Data curation; Formal analysis; Writing‐original draft; Writing‐review & editing. Umesh Rosyara: Conceptualization; Data curation; Formal analysis; Writing‐review & editing. Deepmala Sehgal: Data curation; Writing‐review & editing. Susanne Dreisigacker: Conceptualization; Data curation; Writing‐original draft; Writing‐review & editing.

## CONFLICT OF INTEREST

The authors declare no conflict of interest.

## Supporting information

Supplementary Table S1. List of lines included in this study (see excel file).
Supplementary Table S2. Known genes related to disease resistance and end‐use quality of relevant importance in ESWYT and SAWYT and their approximate physical position.
Supplementary Table S3. Distribution of SNP markers across genomes and the average marker distance in each chromosome.
Supplementary Figure S1. Sequential k clusters with their respective Bayesian Information Criteria (BIC) values.
Supplementary Figure S2. Alpha score statistic for optimal principal components (PC).
Supplementary Figure S3. Density distributions of MAF with their respective thresholds.
Supplementary Figure S4. Minor allele frequency deviation graph from base population (ES1‐7) for every wheat chromosome.

Supporting Information

Supporting Information
