# Supplementary material for: Selection signatures in the CIMMYT International Elite Spring and Semi‐arid Wheat Yield Trials
Source: Plant Genome. 2021 Nov 8;15(1):e20165. doi: 10.1002/tpg2.20165 (PMC12893640; doi:10.1002/tpg2.20165)
Supplement: Supplementary file 1 [file TPG2-15-e20165-s002.docx]

Supplemental Material

**Supplementary Table S1**. List of lines included in this study (see excel file).

**Supplementary Table S2**. Known genes related to disease resistance and end-use quality of relevant importance in ESWYT and SAWYT and their approximate physical position.

| Gene name | Chromosome | Physical position (RefSeq vs 1.1) |
| --- | --- | --- |
| *Glu-A1* | 1A | 508,725,917-508,726,129 |
| *Glu-B1* | 1B | 5,686,560-5,686,783 |
| *Lr46* | 1B | 670,233,646-670,233,769 |
| *Glu-D1* | 1D | 412,162,629-412,163,025 |
| *2NS* | 2A | [3,965,176-3,965,281](https://plants.ensembl.org/Triticum_aestivum/Location/View?r=2A:3965170-3965286;tl=VIYlM1Z0ja7UiuAH-18917837-664026020) |
| *Ppd-A1* | 2A | 36,934,661-36,935,165 |
| *Ppd-B1* | 2B | 56,238,084-56,240,240 |
| *Ppd-D1* | 2D | 33955649-33957800 |
| *Sr2/Fhb1* | 3B | 8,848,496-8,849,092 |
| *Wx-B1* | 4A | 688,099,006-688,099,110 |
| *Rht1* | 4B | 30861349-30861770 |
| *Lr67* | 4D | 221,906,243-221,906,571 |
| *Rht2* | 4D | 18781069-18781438 |
| *Vrn-A1* | 5A | 588550218-588550338 |
| *Vrn-B1* | 5B | 573,815,063-573,815,126 |
| *Vrn-D1* | 5D | 467,183,397-467,183,467 |
| *Pina-D1* | 5D | 3,609,650-3,610,140 |
| *Pinb-D1* | 5D | 3,609,650-3,610,140 |
| *Lr68* | 7B | 740,039,246-740,039,930 |
| *Lr34* | 7D | 47,409,577-47,426,975 |

**Supplementary Table S3**. Distribution of SNP markers across genomes and the average marker distance in each chromosome.

| Wheat Genome | Number of markers |
| --- | --- |
| A Genome | 15290 |
| B Genome | 19878 |
| D Genome | 4848 |
| Unassigned markers | 514 |
| Total | 40530 |
| Chromosome | Average marker distance (bp) |
| 1A | 367646 |
| 1B | 257759 |
| 1D | 659991 |
| 2A | 279346 |
| 2B | 221672 |
| 2D | 876372 |
| 3A | 408103 |
| 3B | 275861 |
| 3D | 689824 |
| 4A | 416747 |
| 4B | 483034 |
| 4D | 1024264 |
| 5A | 381111 |
| 5B | 244176 |
| 5D | 1106551 |
| 6A | 264760 |
| 6B | 227474 |
| 6D | 842443 |
| 7A | 239580 |
| 7B | 241621 |
| 7D | 710536 |

**Supplementary Figure S1.** Sequential k clusters with their respective Bayesian Information Criteria (BIC) values.

**Supplementary Figure S2**. Alpha score statistic for optimal principal components (PC).

**Supplementary Figure S3**. Density distributions of MAF with their respective thresholds.

**Supplementary Figure S4**. Minor allele frequency deviation graph from base population (ES1-7) for every wheat chromosome.
